# Identification of gender-based violence among adult patients in the Emergency Department of Tanzania’s largest public hospital: a retrospective review

**DOI:** 10.1016/j.afjem.2026.100960

**Published:** 2026-03-03

**Authors:** Gimbo Hyuha, Raya Mussa, Daniel Mutani, Juma Mfinanga, Hendry R. Sawe

**Affiliations:** a Emergency Medicine Department, Muhimbili National Hospital, Dar es Salaam, Tanzania; b Department of Social Welfare, Muhimbili National Hospital, Dar es Salaam, Tanzania; c Emergency Medicine Department, Muhimbili University of Health and Allied Sciences, Dar es Salaam, Tanzania

**Keywords:** Gender-based violence, Emergency department, Domestic violence, Intimate partner violence, Rape, Sexual Violence, Emotional Abuse

## Abstract

**Background:**

Emergency Departments (EDs) are critical for identifying and supporting victims of gender-based violence (GBV). However, data from Sub-Saharan Africa are limited, and even in high-income settings with developed EDs, identification remains poor. This study assessed the frequency of GBV identification in the tertiary ED of Tanzania’s largest public hospital and described incident characteristics.

**Methods:**

This retrospective study was conducted in the ED of Muhimbili National Hospital, Tanzania, analyzing patients visits from January 2016 to December 2021. We reviewed electronic medical records of adult patients using a keyword-based search to identify complaints potentially related to GBV. A structured data sheet captured demographics, clinical data, and outcomes. Data were analyzed using Statistical Package for the Social Sciences (SPSS version 25), with descriptive characteristics reported as percentages and means with standard deviations (SD).

**Results:**

Among 296,010 adult ED visits, 87 (0.03%) had GBV-related complaints. The mean age was 32 years (SD 14), and 86% were female. The most common form was sexual abuse (33%), followed by combined sexual and physical abuse (28%). In 21% of cases, a partner was identified as the perpetrator; in 63%, the perpetrator was unknown, refused to disclose or undocumented. Among 61 sexual assault victims, 77% were screened for Human Immunodeficiency Virus (HIV), 49% for syphilis, and 39% for hepatitis. Additionally, 46% received HIV prophylaxis, and 61% of female victims received emergency contraception. Only 14% received psychological consultation. Overall, 63% were admitted, with 84% still hospitalized after 24 h.

**Conclusion:**

GBV identification in this ED was extremely low. This likely reflects a combination of underreporting, low provider awareness, poor documentation, and the limitations of using a retrospective, keyword-based record review. A significant proportion involved sexual violence, with notable gaps in post-assault care. Strengthening GBV screening, management, and follow-up protocols in EDs is urgently needed. Future research should use prospective, robust designs.

**African relevance**

• Gender-based violence (GBV) is a major but under-recognized emergency health crisis across Sub-Saharan Africa.
• Emergency Departments are critical frontline settings yet frequently fail to identify and manage GBV cases effectively.
• This study exposes alarmingly low GBV detection rates in Tanzania’s largest public Emergency Department, highlighting systemic gaps in screening, documentation, and care.
• Urgent action is needed to implement tailored protocols and training to empower African Emergency Healthcare Providerss in responding to GBV survivors.

Alt-text: Unlabelled box dummy alt text

## Introduction

Gender-based violence (GBV) refers to any harmful act or threat of harm directed at individuals based on gender-related power imbalances [1]. GBV encompasses multiple forms of harm such as physical and sexual violence, psychological manipulation, economic domination, and systemic violations including harmful traditional practices such as female genital mutilation. GBV is a global pandemic and a serious violation of human rights. While GBV affects all genders, women and girls are disproportionately impacted with 1 in 3 women experiencing violence in their lifetime [2]. The World Health Organization (WHO) estimates that over a quarter of women aged 15–49 years, who have been in a relationship, have experienced physical and/or sexual violence at least once [2,3].

While the prevalence of lifetime intimate partner violence varies across WHO regions, the African region has one of the highest rates, at 33 %, comparable to the WHO South-East Asia region [2]. In Tanzania, the 2022 Demographic and Health Survey reported that 26 % of women aged 15–49 years have experienced physical violence, and 12 % have experienced sexual violence [4].

Emergency Departments (EDs) are often the first point of contact for patients who have experienced violence, making them crucial for the identification, reporting, treatment, and documentation of GBV cases [5]. EDs encounter multiple forms of GBV, including physical, sexual, emotional, and neglect-related abuse, each requiring tailored assessment and intervention. Interventions can include medical and surgical care, as well as psychological counseling, all provided within the ED. Literature indicates that there is limited data on how GBV is handled in resource-limited EDs, and protocols may vary between institutions; however, most aim to provide comprehensive care, including legal and psychosocial support [2,5].

Reporting of GBV is both critical and a basic patient care need. In high-income countries, where EDs are well established, GBV is often underreported but remains common among patients [5,6]. Emergency medicine is a new and rapidly evolving field in Sub-Saharan Africa (SSA), presenting opportunities for the identification, assessment, and appropriate intervention for GBV. Despite this potential, there is a paucity of data on how well EDs in SSA identify and manage GBV cases. This study aims to describe the frequency, types, and management of GBV cases identified in the ED of Tanzania’s largest public tertiary hospital.

## Methodology

### Study design

This was a retrospective study of all adult patients presenting to the Emergency Department of Muhimbili National Hospital (MNH) in Dar es Salaam, Tanzania, between January 2016 and December 2021.

### Study setting and context

MNH is Tanzania’s national referral hospital with a capacity of 1500 beds. Its ED is the country’s first full-capacity public emergency facility. Patients presenting with gender-based violence (GBV)-related complaints undergo standard triage, followed by multidisciplinary evaluation involving medical personnel, social workers, and, when necessary, police officers. Medical care includes history-taking, physical examination, infection screening and prophylaxis, forensic evidence collection, and consultations for additional services as needed. Social workers document demographics, police report information, and patient history using a structured form. In collaboration with the psychiatry team, psychosocial support is provided. If police have not been notified, social workers initiate reporting and complete special police documentation. Unstable patients are admitted for continued care, while stable patients are discharged with follow-up appointments scheduled within 7–14 days. In some cases, patients are admitted temporarily for protection until police support is secured.

### Study procedures and data abstraction

GBV was defined as any documented instance of physical, sexual, emotional, or neglect-related violence linked to gender-based power dynamics, identified through triage or clinical notes. Gender was recorded as male or female based on biological sex, consistent with Tanzanian legal and cultural norms, as documented in the electronic medical record system (WellSoft™). Emotional abuse was defined as verbal harassment, intimidation, or threats, while neglect included complaints of failure to provide basic needs or protection.

Two trained research assistants with backgrounds in health sciences reviewed all adult ED electronic medical records from January 2016 to December 2021. Data collection occurred between July and September 2022. An initial keyword-based search using terms such as “violence,” “rape,” “sexual abuse,” “neglect,” and “domestic violence” identified potential GBV cases from all records. These cases were then manually reviewed in full. Cases were excluded if the violence was not gender-related or if documentation was insufficient. The manual review process took approximately eight weeks, highlighting both the resource-intensive nature and feasibility limitations of retrospective keyword-based screening.

A structured case report form was used to capture demographics, type and location of violence, perpetrator identity, clinical presentation, investigations performed, management provided, and patient disposition. We also recorded whether sexually transmitted infection (STI) screening was conducted and whether appropriate post-assault care was administered. Documentation quality was assessed, and missing data were noted. Discrepancies were resolved through consensus between the investigators (Gimbo Hyuha and Raya Mussa); however, formal inter-rater reliability testing was not performed.

### Admission and follow-up data

Admission decisions in the department are based on clinical judgment, considering physical injury severity, risk of re-exposure to violence, and mental health status, using a combination of national and international guidelines. No standardized admission criteria exist, which may limit consistency in disposition decisions. Although follow-up appointments were routinely scheduled for discharged patients, data on whether patients attended these appointments or on any longer-term outcomes were not available in the emergency department electronic medical record system and are therefore beyond the scope of this study.

### Outcomes and analysis

The primary outcome was the proportion of adult patients identified with GBV-related complaints in the ED. Secondary outcomes included the type and location of violence, alleged perpetrators, investigations performed, management provided, patient disposition, and 24-hour outcomes. Data were analyzed using SPSS version 25 (IBM Corp., Armonk, NY, USA). The dataset was cleaned, and outliers checked prior to analysis. Descriptive statistics, including means with standard deviations and proportions, were used to summarize the frequency of GBV identification, incident characteristics, clinical findings, and outcomes.

Ethical approval for this study was obtained from the Muhimbili National Hospital Institutional Review Board (*Reference Number:*MNH/IRB/I/2022/062), and permission to publish the findings was granted by the National Institute for Medical Research (*Ref No.* BD.242/437/01C/53). All patient data were fully de-identified and stored securely, with access limited to the study investigators. The study was conducted in accordance with the principles outlined in the Declaration of Helsinki.

## Results

### Patient and incident characteristics

**Fig. 1:**
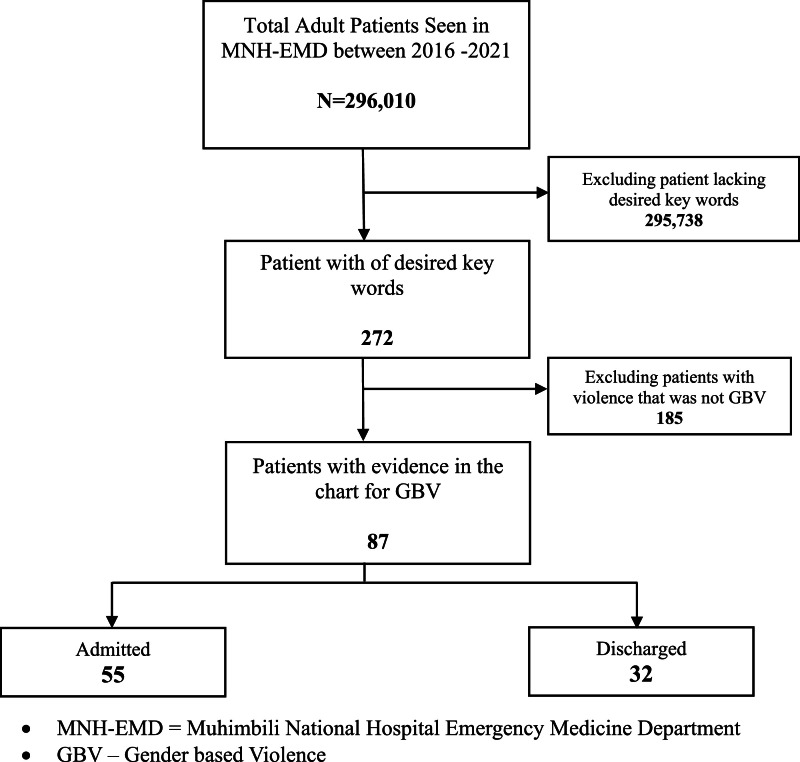
Study flow diagram.

Among 296,010 adults presenting to the ED, 272 (0.1 %) had an incidence of violence documented in their medical record, with 87 (0.03 %) identified as GBV (Fig. 1). The mean age of these patients was 32 years (SD 14), and 86 % were female. The most common form of violence was sexual abuse (33 %). In 21 % of cases, the alleged perpetrator was a partner; in 63 %, the assailant was unknown, refused to disclose, or not documented. Most incidents occurred in public spaces (43 %), with 48 % of patients self-referring and 22 % being brought in by police (Table 1).

**Table 1:** Patient demographics and incident characteristics.

Variable	Mean Years	Standard Deviation (SD)
Age	32	14
	Frequency *N* = 87	Percentage %
Gender		
Female	75	86
Male	12	14
Marital Status		
In relationship	3	3
Married	34	39
Single	42	48
Unknown	4	5
Widowed	4	5
Type of Violence		
Emotional	7	8
Sexual	29	33
Neglect	7	8
Physical	11	13
*Sexual and Emotional	3	3
*Physical and Sexual	24	28
*Emotional and Neglect	1	1
⁎⁎Physical, Sexual and Emotional	5	6
Perpetrator		
Friend	4	5
Other family member or Guardian	10	11
Partner	18	21
Unknown person from streets/thief’s/bar/not mentioned	55	63
Location		
Home	30	34
Others^1^	20	23
Street/Public Places	37	43
Referral		
Brought by Police Officer	19	22
Referred	26	30
Self-Referral	42	48

⁎ Combination of two types of abuse;.

⁎⁎ Combination of three types of abuse;.

1 Includes: Bar, School, Bus stop, Hotel, Night Club Abbreviations: SD = standard deviation.

### ED management and disposition

Among 61 patients reporting sexual assault, 77 % were screened for HIV, 49 % for syphilis, and 39 % for hepatitis. High vaginal swabs for sperm analysis or STI screening were performed in 43 % of female patients, and 61 % were screened for pregnancy (Table 2).

**Table 2:** Emergency Department management and disposition.

Variable	Frequency	Percentage
Sexual assault screening* (*N* = 61)		%
HIV Screening	47	77
Syphilis Screening	30	49
Hepatitis Screening	24	39
Imaging⁎⁎ (*N* = 40)		%
Brain CT Scan	6	15
X Rays of long bones	12	30
No Imaging Done	22	55
CT Findings( (*N* = 6)		
Intracerebral bleeding with/without Fractures	4	67
Normal	2	33
X Ray Findings( (*N* = 12)		
Fracture	2	17
Normal	3	25
No formal radiological report	7	58
Gynecology and Obstetrics Screening(		
High Vaginal Swab (*N* = 54)	23	43
Urinary Pregnancy Test (*N* = 54)	33	61
ED Management		
Emergency Contraceptives (*N* = 54)(	33	61
Gynecology/Urology/Surgical Consultation (*N* = 64)	31	48
HIV Prophylaxis (PEP) (*N* = 61)*	28	46
Psychology Consultation (*N* = 87)	12	14
Intravenous Antibiotics (*N* = 71)	30	42
Analgesics (*N* = 87)	23	26
ED Disposition (*N* = 87)		
Admitted	55	63
Discharged	32	37
24-Hours Outcome (*N* = 55)		
Still admitted	46	84
Discharged	9	16

⁎ Victims of sexual assault;.

⁎⁎ Victims of physical assault;.

η Sexually assaulted female;.

Λ Include the only patients who underwent imaging Abbreviations: HIV = human immunodeficiency virus; PEP = post-exposure prophylaxis; CT = computed tomography; STI = sexually transmitted infection, ED = Emergency Department.

Among 40 patients reporting physical violence, 45 % underwent at least one radiographic investigation:Computer Tomography (CT) scan or X-ray. Of the six who received a head CT, four showed evidences of intracerebral bleeding. Among 12 patients who had long bone X-rays, two were diagnosed with fractures (Table 2).

HIV post-exposure prophylaxis was provided to 46 % of sexual assault victims, and 61 % of females received emergency contraception. Only 48 % of patients requiring consultations (gynecology, urology, or surgery) received them, and just 14 % received psychiatric consultations. Most patients (63 %) were admitted, and 84 % remained hospitalized after 24 h (Table 2).

## Discussion

In this five-year retrospective study, fewer than 1 % of adult patients in our ED were identified as GBV victims. This contrasts sharply with a population-based Tanzanian survey reporting that about 33 % of individuals had experienced GBV [4]. While comparable ED-based data from sub-Saharan Africa are lacking, our detection rate is much lower than those reported in high-income countries (HICs), where GBV, though underreported, is identified more frequently [2,[5], [6], [7]]. Several factors likely contribute to this discrepancy, including the retrospective, keyword-based nature of data collection, limited provider awareness, inadequate screening protocols, and inconsistent documentation. The manual review of 272 flagged cases was resource-intensive and highlights the challenges of retrospective identification, suggesting that the true frequency of GBV in this setting is likely higher. Furthermore, stigma may also cause significant patient under-reporting; for example, a recent Tanzanian survey found low adolescent awareness of GBV health services, correlating with low service utilization [8]. These findings highlight the urgent need to strengthen GBV screening and documentation in emergency care settings, while also enhancing public awareness and accessibility of support services.

Sexual abuse accounted for more than half of all reported forms of violence, which is higher than rates reported in population surveys in Tanzania and other studies from both low- and high-income countries [[4], [5], [6]]. A striking finding was that over half of the individuals affected by GBV were unable or unwilling to identify their perpetrator. Previous research suggests that victims may be reluctant to disclose the perpetrator due to public and familial shaming, threats from the perpetrator, low self-esteem, or fears related to custody issues in cases involving children [9].

One key finding is that over 23 % of sexual assault patients did not receive HIV screening, and fewer than half were screened for syphilis and hepatitis. Only 46 % received HIV prophylaxis and 61 % of eligible females were provided emergency contraception. Additionally, fewer than half of physical assault patients underwent radiological imaging. These gaps highlight the need for comprehensive protocols, staff training, and standard operating procedures to ensure GBV patients receive appropriate screening and post-assault care in limited resource countries.

Regarding disposition, approximately one-third of GBV patients were discharged, while the remainder were admitted, with nearly three-quarters of admitted patients staying over 24 h. Admission decisions were based on clinical judgment. This absence of standardized criteria likely contributes to variability in care. Follow-up appointments were scheduled for discharged patients; however, data on attendance or longer-term outcomes were not available from the ED electronic medical record system and are therefore beyond the scope of this study. These limitations point to the need for robust discharge planning and longitudinal follow-up mechanisms

In Tanzania, GBV is legally recognized as a serious crime, with laws mandating that suspected cases be reported to law enforcement [10]. Victims are entitled to medical care, which includes counseling and social support services. Our ED follows a mix of non-standardized national guidelines and local hospital protocols for the management of GBV cases. These protocols include emergency contraception, HIV prophylaxis, forensic examinations for sexual assault survivors, radiological assessments for physical violence, and psychological support. However, the frequent rotation of medical interns and residents, may affect the consistency of clinicians’ knowledge and adherence to these protocols. If such gaps are evident at Muhimbili National Hospital, Tanzania’s largest public hospital then it is likely that the challenges are even more pronounced in smaller or lower-resourced health facilities across the country.

This study has several limitations. First, as a single-center retrospective analysis with a small sample size, the findings may not be generalizable to other healthcare settings. Relying on keyword searches within the electronic medical record may have led to missed cases due to variability in clinical terminology, documentation practices, patient reluctance to disclose, and inconsistent application of screening protocols. Additionally, the study included only adult patients, potentially excluding a significant number of pediatric GBV cases. Gender was categorized using a binary model, which does not account for individuals who identify as non-binary or gender diverse; however, this approach aligns with the legal and cultural norms in Tanzania. Manual review of flagged cases was resource-intensive and formal inter-rater reliability testing was not conducted. Finally, due to the retrospective design, we could not assess whether admission and discharge decisions were based on clinical judgment or standardized protocols, limiting our understanding of care variability.

Despite these limitations, this study provides important insights into GBV in an ED in Tanzania, highlighting significant gaps in identification, post-assault care, and documentation. Our findings support the urgent need to implement standardized screening protocols, comprehensive training for ED staff, and systematic follow-up to improve survivor outcomes. Future research should include both children and adults, use prospective designs, and track longitudinal outcomes to strengthen evidence for GBV management in low-resource emergency care settings.

## Conclusion

Identification of GBV cases in this ED was extremely low, combination of underreporting, low provider awareness, poor documentation, and the limitations of using a retrospective, keyword-based record review. A substantial proportion of identified cases involved sexual violence, and notable gaps in post-assault care were observed. Strengthening protocols for GBV screening, management, and follow-up in emergency departments is urgently needed to improve survivor outcomes and enhance the health system’s responsiveness to gender-based violence.

## Funding/Support

This project was self-funded.

## Data sharing statement

The dataset supporting this study is available from the author on request.

## Dissemination of results

Findings were shared with the Ministry of Health Tanzanian and respective local government, and members of Emergency Medicine Association Tanzania.

## CRediT authorship contribution statement

**Gimbo Hyuha:** Conceptualization, Methodology, Data curation, Formal analysis, Investigation, Writing – original draft, Writing – review & editing, Supervision. **Raya Mussa:** Conceptualization, Methodology, Formal analysis, Investigation, Writing – original draft, Writing – review & editing, Supervision. **Daniel Mutani:** Methodology, Data curation, Investigation, Writing – review & editing, Project administration. **Juma Mfinanga:** Project administration, Writing – review & editing. **Hendry R. Sawe:** Data curation, Writing – original draft, Writing – review & editing, Project administration.

## Declaration of competing interest

The authors declare that they have no known competing financial interests or personal relationships that could have appeared to influence the work reported in this paper.

## References

[1] UNICEF (2025). Gender-based violence | UNICEF n.d. https://www.unicef.org/protection/gender-based-violence-in-emergencies.

[2] Beyene A.S., Chojenta C., Roba H.S., Melka A.S., Loxton D. (2019). Gender-based violence among female youths in educational institutions of Sub-Saharan Africa: a systematic review and meta-analysis. Syst Rev.

[3] World Health Organization (2024). WHO multi-country study on women’s health and domestic violence against women: summary report n.d. https://www.who.int/publications/i/item/9241593512.

[4] (2024). Tanzania - Demographic and Health Survey and Malaria Indicator Survey 2022 n.d. https://microdata.worldbank.org/index.php/catalog/6102.

[5] Hinsliff-Smith K., McGarry J. (2017). Understanding management and support for domestic violence and abuse within emergency departments: a systematic literature review from 2000 to 2015. J Clin Nurs.

[6] Gupta P.P., Bhandari R., Khanal V., Gupta S. (2018). A cross-sectional study on domestic violence in emergency department of Eastern Nepal. J Fam Med Prim Care.

[7] Boyle A., Todd C. (2003). Incidence and prevalence of domestic violence in a UK emergency department. Emerg Med J.

[8] Mtaita C., Likindikoki S., McGowan M., Mpembeni R., Safary E., Knowledge Jahn A. (2021). Experience and perception of gender-based violence Health Services: a mixed methods study on adolescent girls and young women in Tanzania. Int J Environ Res Public Health.

[9] Muluneh M.D., Stulz V., Francis L., Agho K. (2020). Gender based violence against women in Sub-Saharan Africa: a systematic review and meta-analysis of cross-sectional studies. Int J Environ Res Public Health.

[10] Anitha R. (2023). An appraisal of the legal obligations vested to Tanzania and the legal challenges facing the prohibition of gender-based violence in Tanzania. J Leg Stud Res.

